# Olaparib induced aplastic anemia in a patient with castrate resistant prostate cancer: A case report

**DOI:** 10.1016/j.lrr.2024.100473

**Published:** 2024-07-24

**Authors:** Elrazi A Ali, Monika Jain, Akriti Pokhrel, Unni Mooppan, Jen chin Wang

**Affiliations:** a Internal Medicine Department, Interfaith Medical Center, One Brooklyn Health, Brooklyn, NY, USA; b Department of Hematology and Medical Oncology, Brookdale University Hospital Medical Center, One Brooklyn Health, Brooklyn, NY, USA; c Department of Urology, Brookdale University Hospital Medical Center, One Brooklyn Health, Brooklyn, NY 11212, USA

**Keywords:** Aplastic anemia, Olaparib, Prostate cancer, Pancytopenia

## Abstract

Olaparib is (ADP‐ribose) polymerase inhibitor (PARPi), which stops the repair of single-stranded DNA breaks. This leads to the death of cancer cells with BRCA1/BRCA2 mutations or homologous recombination deficiency. Since being approved by the FDA in 2023 for treating castrate-resistant prostate cancer (CRPC), there have been some reports of myelodysplastic syndrome (MDS) and acute leukemia linked to PARP inhibitor use for ovarian, breast, pancreatic and breast cancers, there have been no reports of aplastic anemia after receiving PARPi therapy. This case report describes a 75-year-old man with BRCA2-positive metastatic castrate-resistant prostate cancer who developed aplastic anemia after taking olaparib.

## 1 Introduction

Many tumors carry defective genes involved in DNA repair mechanisms; as a result, they can be targeted by antineoplastic treatment. Olaparib is an antineoplastic agent that was first approved by the FDA in Dec 2014 for treating advanced ovarian cancer. It was the first of its class to be used in clinical practice. The drug is used for treating breast, ovarian, pancreatic, and prostate cancer [1]. Olaparib works by inhibiting poly (ADP‐ribose) polymerase (PARP) 1 and 2 that prevents the repair of single-stranded DNA breaks. This results in the death of BRCA cancer cells that have dysfunction in DNA repair [2]. In BRCA mutated cells, DNA will be repaired via error-prone processes, including single-strand annealing and non-homologous end joining; when there is a lot of DNA damage, these alternative repair processes will be overwhelmed, resulting in cell death. During clinical trials, the drug was discontinued due to various side effects. The most frequent adverse effects reported in clinical trials were gastrointestinal nausea, vomiting, diarrhea, dyspepsia, also tiredness, headache, and an increase in baseline serum creatinine levels in more than 10 % of patients [2]. Also, dizziness, arthralgia, and back pain were not uncommon. Hematologic adverse effects include anemia, thrombocytopenia, and neutropenia (>10 %). However, there have been no reports of aplastic anemia as per our review of the English literature. We are reporting an interesting case of olaparib-associated aplastic anemia in a 75-year-old man with BRCA2-positive metastatic castrate-resistant prostate cancer.

## 2 Case presentation

**Fig. 1:**
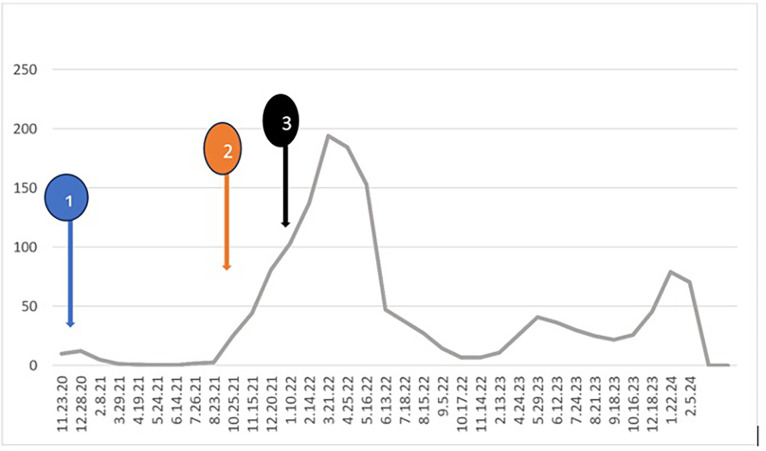
The trend of PSA level in ng/mL (vertical axis) and the treatment received during that period: 1: started on Leuprolide, Abiraterone and Docetaxel, 2: switched to cabazitaxel abiraterone, 3: switched to olaparib, abiraterone.

**Fig. 2:**
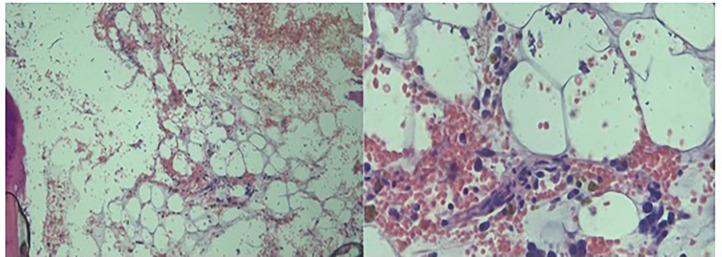
Markedly hypocellular fatty bone marrow with 5 to 10 % cellularity with normal trilineage hematopoietic elements showing adequate maturation.

A 75-year-old male was diagnosed with prostate cancer in 2020, with prostate biopsy showing high Gleason score [8,9] in multiple lobes. Prostate-specific antigen (PSA) at diagnosis was 10.2 ng/mL. Staging with positron emission tomography (PET) scan showed sclerotic bone metastases on the right femur head, left 9th and 10th rib, right and left 7th rib, left pubic symphysis, and vertebral metastases in the lower thoracic and lumbar spine. A whole body bone scan showed multiple bony metastases: in the right humeral head region, multiple ribs bilaterally, the vertebral spine (more prominent in the low thoracic and lumbar region), pelvic bones bilaterally, left proximal femur. PET Scan showed mild diffuse heterogeneous hypermetabolic activity throughout the bone marrow and multiple sclerotic bone metastases throughout the skeleton. He was initially treated with triple therapy consisting of Leuprolide, Abiraterone, and Docetaxel. After triple therapy, PSA dropped, and then uptrended again over the time. Pt was then given cabazitaxel and abiraterone. PSA was rising with cabazitaxel and abairaterone so was switched to Olaparib (initially 300 mg bid then reduced to 200 mg bid) and abiraterone in January 2022. Response in PSA with various medications given is shown in a timeline graph in Fig. 1. Pt was receiving Leuprolide all along although other medications were being switched. Pt was doing well, but again PSA appeared to be slightly uprising along with laboratory finding of mild cytopenia. Olaparib was discontinued in September 2023. When Lutetium -177 PSMA therapy was in the process of getting approved by insurance, in February 2024, he presented to the emergency department with fever, chills, malaise, fatigue, dizziness, and epistaxis. Laboratory findings showed severe pancytopenia with nadir of hemoglobin 3.4 g(g)/ deciliter(dl), white blood cells (WBC) 1.2 × 10^3^/microliter(uL) and platelets 3 × 10^3^/uL. Bone marrow biopsy (Fig. 2) showed markedly hypocellular fatty bone marrow showing 5 to 10 % cellularity with normal trilineage hematopoietic elements showing adequate maturation. No atypical infiltrate was seen. Iron stain 3+. Flow cytometry showed no evidence of an increased blast population or a lymphoproliferative disorder. Anti neutrophil antibody (ANA), rheumatoid factor(RF), and anti-DNAse antibody titer were negative. Work up suggested aplastic anemia. He received multiple blood product transfusions, including approximately thirty units of PRBCs, thirty units of platelets and 6 doses of filgrastim over a period of 32 days. He had been on Abiraterone, and Lupron, continuously for 2 years; Olaparib was discontinued a few months ago when pancytopenia discovered. Patient was in the process of getting approval for using antithymocyte globulin (ATG), cyclosporin and Eltrombopag, (an oral thrombopoietin-receptor agonist). Unfortunately, he had a mechanical fall complicated by intracranial hemorrhage and expired .

## 3 Discussion

Aplastic anemia (AA) is defined as pancytopenia in combination with bone marrow hypoplasia or aplasia in the absence of an abnormal infiltrate or marrow fibrosis [3]. The acellular bone marrow is the cornerstone in the diagnosis to differentiate AA from other causes like hypersplenism or infiltrative disorders. AA can be caused by several disorders, commonly autoimmune disorders, exposure to toxins, viral infections (such as hepatitis, Epstein-Barr virus, or HIV), genetic predisposition, and cytotoxic drugs [4].

Olaparib was approved by the FDA on May 19, 2020, for adult patients with metastatic castration-resistant prostate cancer with deleterious germline or somatic homologous recombination repair (HRR) gene mutations. Then, in May 31, 2023 FDA approved Olaparib along with abiraterone and prednisone (or prednisolone) for treating patients with deleterious or suspected deleterious BRCA-mutated mCRPC. Subsequently, other PARP inhibitors combination Talazoparib + Enzalutamide, Niraparib + Abiraterone Acetate + Prednisone, were approved in 2023 for treating patients with metastatic castration-resistant prostate cancer with alterations in BRCA1, BRCA2, and HRR deficiency gene mutation [5]. Olaparib is associated with extended radiographic progression-free survival and overall survival in this population [6].

Many studies have tried to cover the side effects of olaparib . According to further analyses from PROfound study, which used Olaparib in patients with metastatic CRPC, the most frequent side effects were nausea (41 %), anorexia (30 %), and fatigue (41 %), with anemia (46 %) being the most common hematologic side effect [6]. These side effects were low-grade and were managed without the need to discontinue treatment [6]. Other less common hematologic side effects include isolated thrombocytopenia or neutropenia in patients [7]. Rare side effects include reports of acute leukemia, which is reported to occur after a long duration of treatment of 1.5 years [8,9]. One study compared the incidence of MDS and acute leukemia in patients with ovarian cancer. It showed that there was overall no difference in MDS/AML in the olaparib and placebo groups (2.1 % vs 4.0 %) over a follow-up period of 25 months. In both groups, the risk was high post-platinum treatment [10]. However, post-marketing studies showed an increased risk. One large study showed a significantly increased risk after latency of MDS and AML associated with PARP inhibitors, with 211 days for MDS and 355 days for AML [11]. With mortality, MDS and AML caused by the four PARP inhibitors were 39.23 and 45.39 %, respectively. Another cohort showed that AML following olaparib is associated with poor prognosis and survival due to unfavored cytogenetics [12]. The reported AML and MDS with olaparib were seen in a few case reports and mainly on patients with prolonged follow-ups [13]

There are other several studies that have assessed olaparib in various solid cancers, but only one previous study had mentioned side effect of aplastic anemia. In PAOLA-1 trial, patients on Olaparib with bevacizumab were reported to have MDS/ AML/ aplastic anemia as side effects. In 1 % of patients receiving maintenance olaparib plus bevacizumab vs. 0.4 % of placebo plus bevacizumab patients these side effects i.e. MDS or AML or aplastic anemia was seen [14,15]. However PAOLA-1 trial doesnot specify how many of those 1 % or if any were aplastic anemia.

Hematological toxicity of PARP inhibitors is usually reported to occur early during the treatment, as the FDA adverse event reporting system (FAERS) reporting system has shown (reports from 2015 to 2021) [16]. The median time for hematologic toxicity was around 28 days [10], with mortality reaching 8.76 compared to other PARPi; the time of onset of hematologic toxicity of olaparib was longer with a median onset of 63 days [16] and was shortest of 21 [9] days for niraparib. Hematological toxicity of PARP inhibitors are associated with considerable high mortality. Among PARP inhibitors, olaparib was reported to have the highest fatality proportion, reaching 16.3 %, followed by talazoprib at 15.89 % [16]. Our patient was started on olaparib from January 2022 till September 2023, when he developed pancytopenia with WBC, 2.09 x10^3^/uL, Hb of 9 g/dl, and platelet counts of 80- x10^3^/uL.

AA is a very rare adverse event of olaparib. The mechanism that can contribute to olaparib related aplastic anemia is not clear. The mechanism by which olaparib causes aplastic anemia can be one of two major mechanisms. Firstly, aplastic anemia can occur in a dose-dependent form; some medications are associated with aplastic anemia at higher doses. These substances can directly damage the bone marrow, leading to suppression of blood cell production. In this case, the severity of aplastic anemia may correlate with the dose and duration of exposure to the drug. For example, cyclophosphamide, methotrexate, and chlorambucil are associated with dose-dependent bone marrow suppression. Secondly, idiosyncratic reaction at standard therapeutic doses. In these cases, the development of aplastic anemia may be influenced by individual susceptibility factors and immune responses. Like chloramphenicol, methimazole and carbamazepine. There is minimal data on whether aplastic anemia associated with olaparib is idiosyncratic or dose-dependent, related to chronic double-strand DNA synthesis inhibition [2]. Our patient developed aplastic anemia with the usual dose, which is probably an idiosyncratic reaction.

Our thorough literature review in the articles available in the English literature on olaparib did not show any specified mechanism . It is probably related to olaparib's own mechanism of action. While this mechanism is beneficial in targeting cancer cells, it can also affect normal cells, including hematopoietic stem cells in the bone marrow. With prolonged exposure, olaparib may lead to the accumulation of DNA damage in these stem cells, potentially disrupting their function. The likely mechanism is that if the defective DNA repair mechanism results in the death of all stem cells, then AA will appear, and if the defective repair lead to mutations and these stem cell survive, then these could result in AML or MDS.

Despite the profile with many side effects, olaparib prevent**s** deterioration in health-related quality of life scores and was associated with a reduced pain burden over time compared to other PARP inhibitors in patients with prostate cancer [7].

## 4 Conclusion

Aplastic anemia is a serious and uncommon side effect of olaparib with the regular maintenance dose. We believe we are reporting the first case of Olaparib-induced aplastic anemia. An earlier discontinuation of Olaparib treatment may be worthwhile to be investigated.

## Ethics approval

Ethics approval to report this case was obtained from Brookdale Hospital Institutional Review Board. Our institution does not require ethical approval for reporting individual case reports. (Verbal consent was obtained before patient was diseased).

## Declaration of generative AI and AI-assisted technologies

The authors declared that they did not use AI technology to write the manuscript.

## CRediT authorship contribution statement

**Elrazi A Ali:** Data curation, Investigation, Writing – review & editing, Writing – original draft. **Monika Jain:** Data curation, Writing – review & editing. **Akriti Pokhrel:** Conceptualization, Data curation, Investigation, Writing – review & editing. **Unni Mooppan:** Data curation, Investigation. **Jen chin Wang:** Conceptualization, Data curation, Funding acquisition, Methodology, Writing – review & editing.

## Declaration of competing interest

Jen C Wang, MD received research grant from Kartos incorporation, The other author(s) declared no potential conflicts of interest.

## References

[1] de Bono J., Mateo J., Fizazi K., Saad F., Shore N., Sandhu S., Chi K.N., Sartor O., Agarwal N., Olmos D., Thiery-Vuillemin A. (2020). Olaparib for metastatic castration-resistant prostate cancer. N. Engl. J. Med..

[2] Goulooze S.C., Cohen A.F., Olaparib Rissmann R. (2016). Br. J. Clin. Pharmacol..

[3] Cuglievan B., DePombo A., De Angulo G. (2016). Aplastic anemia: the correct nomenclature matters. Haematologica.

[4] Young N.S. (2002). Acquired aplastic anemia. Ann. Intern. Med..

[5] Fallah J., Xu J., Weinstock C., Brave M.H., Bloomquist E., Fiero M.H., Schaefer T., Pathak A., Abukhdeir A., Bhatnagar V., Chiu H.J. (2024). FDA approval summary: olaparib in combination with abiraterone for treatment of patients with BRCA-mutated metastatic castration-resistant prostate cancer. J. Clin. Oncol..

[6] Roubaud G., Özgüroğlu M., Penel N., Matsubara N., Mehra N., Kolinsky M.P., Procopio G., Feyerabend S., Joung J.Y., Gravis G., Nishimura K. (2022). Olaparib tolerability and common adverse-event management in patients with metastatic castration-resistant prostate cancer: further analyses from the PROfound study. Eur. J. Cancer.

[7] Thiery-Vuillemin A., de Bono J., Hussain M., Roubaud G., Procopio G., Shore N., Fizazi K., Dos Anjos G., Gravis G., Joung J.Y., Matsubara N (2022). Pain and health-related quality of life with olaparib versus physician's choice of next-generation hormonal drug in patients with metastatic castration-resistant prostate cancer with homologous recombination repair gene alterations (PROfound): an open-label, randomised, phase 3 trial. Lancet Oncol..

[8] Zhu J., Tucker M., Wang E., Grossman J.S., Armstrong A.J., George D.J., Zhang T. (2017). Acute myeloid leukemia after olaparib treatment in metastatic castration-resistant prostate cancer. Clin. Genitourin. Cancer.

[9] Liu X., Wang E. (2018). 161 therapy-related acute erythroid leukemia after olaparib treatment. Am. J. Clin. Pathol..

[10] Korach J., Turner S., Milenkova T., Alecu I., McMurtry E., Bloomfield R., Pujade-Lauraine E. (2018). Incidence of myelodysplastic syndrome (MDS) and acute myeloid leukemia (AML) in patients (pts) with a germline (g) BRCA mutation (m) and platinum-sensitive relapsed ovarian cancer (PSR OC) receiving maintenance olaparib in SOLO2: impact of prior lines of platinum therapy. JCO.

[11] Zhao Q., Ma P., Fu P., Wang J., Wang K., Chen L., Yang Y. (2022). Myelodysplastic syndrome/acute myeloid leukemia following the use of poly-ADP ribose polymerase (PARP) inhibitors: a real-world analysis of postmarketing surveillance data. Front. Pharmacol..

[12] Marmouset V., Decroocq J., Garciaz S., Etienne G., Belhabri A., Bertoli S., Gastaud L., Simand C., Chantepie S., Uzunov M., Genthon A. (2022). Therapy-related myeloid neoplasms following PARP inhibitors: real-life experience. Clin. Cancer Res..

[13] Morice P.M., Leary A., Dolladille C., Chrétien B., Poulain L., González-Martín A., Moore K., O'Reilly E.M., Ray-Coquard I., Alexandre J. (2021). Myelodysplastic syndrome and acute myeloid leukemia in patients treated with PARP inhibitors: a safety meta-analysis of randomised controlled trials and a retrospective study of the WHO pharmacovigilance database. Lancet Haematol..

[14] Morice P.M., Leary A., Dolladille C., Chrétien B., Poulain L., González-Martín A., Moore K., O'Reilly E.M., Ray-Coquard I., Alexandre J. (2021). Myelodysplastic syndrome and acute myeloid leukaemia in patients treated with PARP inhibitors: a safety meta-analysis of randomised controlled trials and a retrospective study of the WHO pharmacovigilance database. Lancet Haematol..

[15] Ray-Coquard I., Leary A., Pignata S., Cropet C., González-Martín A., Marth C., Nagao S., Vergote I., Colombo N., Mäenpää J., Selle F. (2023). Olaparib plus bevacizumab first-line maintenance in ovarian cancer: final overall survival results from the PAOLA-1/ENGOT-ov25 trial. Ann. Oncol..

[16] Shu Y., Ding Y., He X., Liu Y., Wu P., Zhang Q. (2023). Hematological toxicities in PARP inhibitors: a real-world study using FDA adverse event reporting system (FAERS) database. Cancer Med..

